# B for Beethoven

**DOI:** 10.3201/eid2909.AC2909

**Published:** 2023-09

**Authors:** Terence Chorba

**Affiliations:** Centers for Disease Control and Prevention, Atlanta, Georgia, USA

**Keywords:** Ludwig van Beethoven, art science connection, emerging infectious diseases, art and medicine, about the cover, viruses, B for Beethoven, hepatitis B virus, HBV, Paget’s disease, Portrait of Beethoven, 1823, Ferdinand Georg Waldmüller

**Figure:**
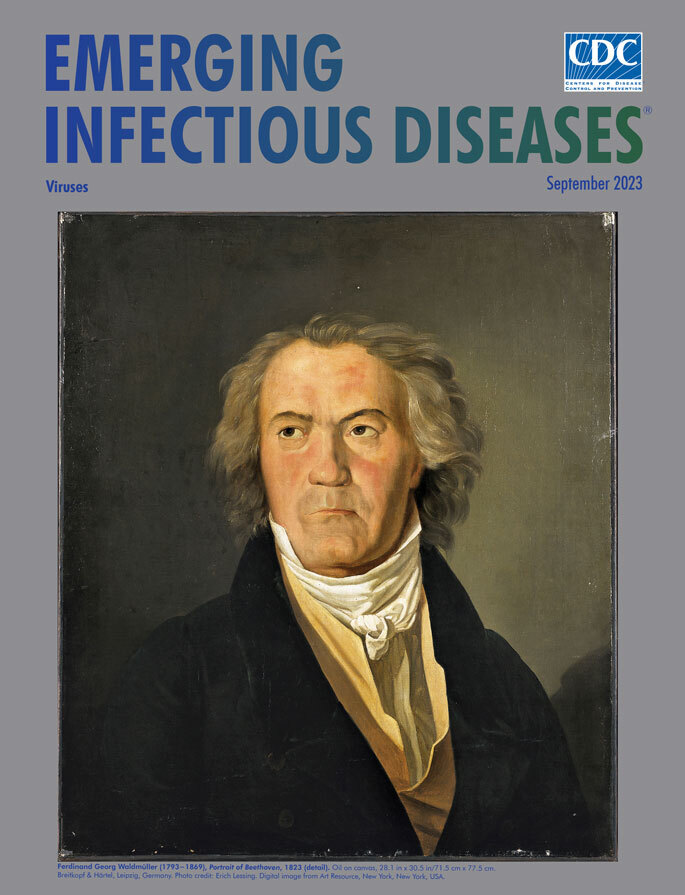
**Ferdinand Georg Waldmüller (1793−1869), *Portrait of Beethoven*, 1823 (detail).** Oil on canvas, 28.1 in x 30.5 in/71.5 cm x 77.5 cm. Breitkopf & Härtel, Leipzig, Germany. Photo credit: Erich Lessing. Digital image from Art Resource, New York, New York, USA

Ludwig van Beethoven (1770‒1827) is one of the most renowned and admired composers in the development of Western Classical music. He was perhaps the greatest contributor to the musical style transition from Classical (roughly 1750‒1820), with linear compositional styles, to Romantic (roughly 1798‒1837), with dramatic expansion of orchestra size and development of lyrical, less formulaic melodic styles. The German composer’s contributions vastly widened the scope and development of the concerto, quartet, sonata, and symphony. In March 1827, after a prolonged illness, Beethoven died at age 56 in his apartment in Vienna. Discussions of Beethoven’s health have been voluminous, fraught with controversy, and limited by an absence of evidence, characteristic of the first half of the 19th century before the availability of radiologic and microbiologic diagnostics. Starting at age 28, the composer suffered hearing deficits that were initially characterized as tinnitus and high-frequency hearing loss. Letters, journals, and other documents of that era indicate that, in his final decade of his life, Beethoven’s health and hearing progressively declined, yet he produced many works that were expansive and departing from the more conservative structure of his earlier works.

In 1823, Ferdinand Georg Waldmüller (1793‒1865), a Vienna-born painter, was commissioned by Christoph Härtel, one of Beethoven’s Leipzig publishers, to paint a portrait of the composer. Waldmüller is credited with being one of the most influential painters of the Biedermeier period, the era between the Congress of Vienna in 1815 and the onset of the revolutions throughout Europe in 1848. Beginning at age 14, Waldmüller studied portrait, still life, and nature painting at the Academy of Fine Arts in Vienna. In the painting featured on this month’s cover, he captured an older Beethoven whose hair, though still wild, was a bit more tame than in images from his younger years. Beethoven was already suffering from hearing loss, but that was the same year in which he completed one of his supreme achievements, his Missa Solemnis (https://archive.org/details/lp_missa-solemnis_ludwig-van-beethoven-leonard-bernstein-the). The composer sat for Waldmüller only once and that sitting was brief, so it is assumed that only the composer’s face was captured; later on, the painter would have added the clothes and portions of the hair. A second, more finished, oil-on-canvas version of the portrait was made from that study but was destroyed in a fire during the 1943 Allied bombing of Leipzig. Later in his career, Waldmüller focused on painting landscapes; his most notable works, principally in Italy, emphasized nature and color. He died in 1865 in Hinterbrühl, Austria, near his native Vienna.

The day after Beethoven’s death, an autopsy performed by one of the leading pathologists of the era, Karl Rokitansky, found Beethoven to have a uniformly dense skull vault; together with Beethoven’s prominent forehead and enlarged jaw with protruding chin, that finding is thought to have been consistent with Paget’s disease of bone (osteitis neoformans), not described until 1877. Paget’s is a disease of unknown etiology in which there is cellular remodeling and bone deformity from breakdown and disorganized new bone formation. Progressive hearing loss is a common symptom of Paget’s disease, the result of the eighth cranial nerve being compressed by bony overgrowth or the small bones of the middle ear being disrupted. Another autopsy finding, an atrophic nodular and cirrhotic liver, together with the account of a friend that Beethoven consumed wine in excess near the end of his life, has long led historians to believe that Beethoven also suffered from and died of alcoholism-associated liver disease. There is no identified record of his having palmar erythema, spider angiomata, asterixis (liver flap), or gynecomastia, all commonly associated with chronic liver disease; however, Beethoven endured 2 attacks of jaundice (the first at age 51), swelling of his limbs, and ascites requiring repeated paracentesis. In most cases worldwide, cirrhosis of the liver is attributable to the interplay of individual genetic predisposition and the effects of alcohol or infection with hepatitis B virus (HBV) or hepatitis C virus (HCV). 

In a fortuitous recent development in the study of the human genome, hair has been identified as a potential resource for evidence of HBV DNA in persons with acute or chronic HBV infection. Recently, 8 independently sourced locks of hair attributed to Beethoven from public and private collections underwent genomic sequencing, 5 of which we now know originated from the same man with predominantly central European ancestry and are deemed to be authentic. DNA extracted from those 5 locks yielded 2 copies of a particular variant of the *PNPLA3* gene that has been associated with developing liver cirrhosis. The 5 locks also had single copies of 2 variants of the *HFE* gene that most often cause hereditary hemochromatosis, which can also contribute to liver damage. Based on metagenomic analyses, it seems that Beethoven also had HBV infection, at least in the months immediately before his death, although it is unknown whether the infection was recent, chronic, or reactivated. Thus, a potential explanation for Beethoven’s recurrent bouts of jaundice and the severe liver disease observed at autopsy, often credited as his principal cause of death, may be the contribution of any or all of the triad of excess alcohol consumption, genetic predisposition for liver disease, and HBV infection. 
